# Simulation of visual acuity by personalizable neuro-physiological model of the human eye

**DOI:** 10.1038/s41598-019-44160-z

**Published:** 2019-05-24

**Authors:** Csilla Fülep, Illés Kovács, Kinga Kránitz, Gábor Erdei

**Affiliations:** 1 Department of Atomic Physics, Budapest University of Technology and Economics, H-1111 Budapest, Hungary; 2 Department of Ophthalmology, Semmelweis University, H-1085 Budapest, Hungary; 3 Department of Ophthalmology, Weill Cornell Medical College, New York City, NY, USA

**Keywords:** Image processing, Biological physics

## Abstract

We present a model of the whole visual train to estimate an individual’s visual acuity based on their eye’s physical properties. Our simulation takes into account the optics of the eye, neural transmission and noise, as well as the recognition process. Personalized input data are represented by the ocular wavefront aberration and pupil diameter, both either coming from *in vivo* measurements of a subject or being produced by optical design software using a schematic eye. This flexibility opens the door to a broad range of potential applications, such as objective visual acuity measurements and intraocular lens design. Our algorithm contains only two adjustable neural parameters: additive noise *σ*, and discrimination range *δρ*, with their values being experimentally calibrated by fitting the results of simulations to the outcome of real acuity tests performed on healthy young subjects with normal vision (visual acuity: 0…−0.3 logMAR range). It was established that by using fixed values of *σ* = 0.10 and *δρ* = 0.0025 for each person examined, the residual of the acuity simulations averaged over the calibration group reached its minimum at 0.045 logMAR.

## Introduction

Visual acuity is the single most important ophthalmological quantity describing the perceived resolving power of the human eye. Conventional acuity tests are performed using eye charts, for which purpose the Early Treatment Diabetic Retinopathy Study (ETDRS) chart, implemented with the Sloan characters, has become the standard^1–3^. In such tests, the subject’s task is to correctly recognize letters of different scales, where letter size is characterized by the visual angle *α*, of the stroke width. In line with the sensitivity of the human eye to lowering stimulation, in eye charts currently used the letter size decreases from line to line in a geometric progression with a ratio of 10^1/10^ , ^3–5^. Thus, the visual acuity value *V*, is usually expressed in logMAR units (i.e. the decimal-base logarithm of the Minimum Angle of Resolution) such as:

1 $$V\equiv {\log }_{10}({\alpha }_{0}),$$

where *α*_0_ denotes the visual angle in minutes of arc at the 50% recognition probability threshold by definition of the International Council of Ophthalmology (ICO) standard^1,2^. This measurement process is used to assess the entire visual system, as a result of which the acuity value depends not only on the optical parameters of the human eye, but is affected by factors such as retinal sampling, neural transfer, neural noise, and cortical recognition^6,7^. Accordingly, reliable visual acuity models should accurately take all these phenomena into consideration^7–10^.

**Figure 1:**
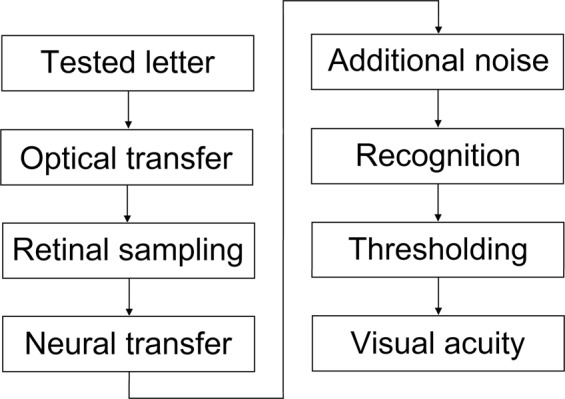
The outline of visual acuity models presented in the literature.

The primary goal of vision models is to relate precisely measurable objective optical/mechanical parameters to the subjective visual acuity value^7,10^. In order to establish such a relationship, first the image quality of the eye has to be known. The “optically filtered” retinal image can be reconstructed from the monochromatic aberrations of the human eye^11–14^, which is measurable using Shack-Hartmann wavefront sensors^15^. As a next step, neural transfer simulates the post-retinal neural image, with additional neural noise being taken into account to determine the noisy image which is finally recognized by the visual cortex^16,17^. The recognition process is usually represented as a template-matching algorithm, which is known to be one of the simplest and oldest models of pattern vision^18–20^. By examining the correct/incorrect identifications of several letters of a given size, it is possible to estimate the recognition probability from one line to the next. Vision models use these data to calculate the psychometric function of vision by curve-fitting, from which the visual acuity value can be determined by thresholding^7,9^. The outline of a typical visual acuity model is depicted in Fig. 1.

Most visual acuity models analyze optical aberrations and diffraction effects through the use of the monochromatic Point Spread Function, *PSF*. More elaborate implementations take into account the severe longitudinal chromatic aberration of the eye^11,21,22^ by integrating defocused *PSF*s for a few discrete monochromatic wavelengths^10,21^. However, we found that this was neither sufficiently precise nor very practical. Some other models utilize Bayesian probability theory to provide a more realistic description of recognition^10,21,23^, which increases simulation time, but does not address the individual characteristics of optical transfer. Another possibility is to model recognition with neural networks^24,25^, which has become widespread recently— especially for identifying handwritten digits^26,27^ or characters^28–30^. The main drawback of using artificial intelligence is that a huge number of samples are required during the training phase.

Given the stated limitations, we decided to develop a new personalizable visual acuity model based on those propounded in the literature^7,9,10^. Since most medical treatments attempt to restore the patients’ vision capability to a normal level, we focused our attention on studying the visual acuity of healthy people with near-emmetropic vision, examining only the ±0.5° field angle range of foveal vision. Our intention was to create a simple simulation tool having as few parameters as possible, in which one can modify the opto-mechanical structure and analyze the resulting effects. Thus, we implemented a physiologically correct schematic eye that can accurately describe the polychromatic imaging properties of any specific human eye, to which we added a simplified but realistic representation of the retina. We supplemented it with an improved numeric model of neural processes and cortical recognition, and integrated a novel algorithm that estimates the visual acuity value. The specific motivation for our research came from the field of cataract surgery: we were particularly interested in improving the design, measurement, and implantation of intraocular lenses (IOLs). Our practice shows that the comparison of different IOL types (e.g. aspheric, toric, or diffractive lenses) and also the evaluation of alternative designs of the same type would greatly benefit from a simulation tool that estimates post-operative visual acuity^31,32^. Moreover, the same model could be used to devise new visual acuity measurements to objectively assess the outcome of surgery^33,34^. Such objectives may not be universally required, but we expect that our model will prove useful for other areas of vision research, too.

In this paper, we present our model in detail and demonstrate its capability to algorithmically reconstruct the monocular visual performance of a small group of young subjects based on the measured optical properties of their eyes. Furthermore, we provide preliminary calibration values for the two adjustable neural parameters of the model. Sensitivity to changes in different construction parameters are discussed and a comparison with the former Watson-Ahumada model^7^ is outlined.

## Methods

### Basics of vision modeling: image formation and neural processing

Visual acuity models presented in the literature^7,9,10^ determine the generalized (complex) pupil function *O*(*X*, *Y*), using a simplified formula derived from wavefront aberration (i.e. Optical Path Difference, *OPD*):

2 $$O(X,Y)=T(X,Y)\cdot \exp (-i\cdot 2\pi \cdot OPD(X,Y)),$$

where *T*(*X*, *Y*) describes the amplitude transmission of the pupil, *X* and *Y* denote coordinates on the exit pupil of the eye, and *OPD* is given in wavelength units of *λ*_0_ (the reference wavelength, e.g. that of the aberrometer). From *O*(*X*, *Y*), the *PSF* can be computed as the squared modulus of its Fourier transform. It should be noted that it is very common to formulate the wave function as if the phase *advances* in the direction of wave propagation. Since we follow this sign convention, in our case the Huygens-Fresnel diffraction integral specifies a Fourier transform and not its inverse.

Since biological visual systems are considered to be linear in terms of incoherent irradiance at the retina^27^, the foveal image of an arbitrary object can be obtained by convolving the ideal (paraxial) image with the *PSF*. As spatial domain convolution is equivalent to multiplication in the frequency domain, the calculations are usually implemented in the latter form^35^. In this way, the Optical Transfer Function, *OT F*, (being the inverse Fourier transform of the *PSF*) is used to characterize the optical system^12^. Consequently, the irradiance distribution *RI*(*x*, *y*), of the image at the retina can be calculated as:

3 $$RI(x,y)={\rm{F}}{\rm{T}}\,[{\rm{I}}{\rm{F}}{\rm{T}}[II(x,y)]\cdot OTF({f}_{x},{f}_{y})],$$

where *II*(*x*, *y*) indicates the ideal image (magnified image of the object), while FT and IFT stand for Fourier transform and its inverse, respectively. In order to avoid any confusion that may arise from expressing the optical image of eyes having different focal length by spatial coordinates at the retina, we project the image back into the object space and present coordinates in angular units (*x* and *y* are expressed in degrees). Accordingly, *f*_*x*_ and *f*_*y*_ indicate angular frequencies in cycles/degree.

In visual acuity models, the optical transfer is followed by retinal sampling that characterizes the effects of the photoreceptor mosaic. According to anatomical studies, cones are arranged in a quasi-hexagonal lattice with approximately 50…60 cycles/degree Nyquist limit at the fovea centralis^10,17,36^. Therefore, the sampling process can be modeled using a low-pass filter whose prime function is to decrease spatial resolution. Nevertheless, according to previous experimental findings^10,21^, this effect is only significant for almost aberration-free, diffraction limited eyes; whilst in the case of average vision it is negligible.

There are certain models that take into account the contrast drop caused by light scattering that occurs at the cornea and the crystalline lens. Since at this phase of the project we deal only with healthy young subjects whose vision is affected by scattered light only in a minor extent^37^, for the time being we do not take it into consideration.

As a next step, the Neural Transfer Function, *NTF*, should be incorporated to model low-level retinal image processing. It can be either measured directly without optical effects, bypassing the optics by interferometric techniques, or derived from the Contrast Sensitivity Function, *CSF, *^7,9^. The *CSF* is a radially symmetric band-pass filter composed of a low and a high-frequency lobe, from which low-pass filtering corresponds to convolution with a blurring mask^21,28,29^. Since in the frequency domain overall contrast is the product of the optical and neural filters, the *NTF* can be determined by dividing the *CSF* by the Mean Optical Transfer Function, *MOTF*:

4 $$NTF=\frac{CSF}{MOTF}.$$

The most accurate characterization of the *MOTF*— which represents the average optical transfer function of the best-corrected human eye— is presented by Watson^38^. Applying this, the shape of the resulting *NTF* curve corresponds to that of edge-enhancement filters. Analogously to optical filtering, the post-retinal neural image *PI*(*x*, *y*), can be expressed as:

5 $$PI(x,y)={\rm{F}}{\rm{T}}[{\rm{I}}{\rm{F}}{\rm{T}}[RI(x,y)]\cdot NTF({f}_{x},{f}_{y})].$$

The mathematical construction *PI*(*x*, *y*) represents the electrical signals that the retina sends towards the visual cortex. This takes us to the last step of image processing: the calculation of a noisy image, that is to be analyzed by the cortical recognition process. As with all biological organs, the visual system also has some temporal uncertainty^16,17^, which is usually modeled at one instant as additive noise:

6 $$NI(x,y)=PI(x,y)+GWN(x,y).$$

In (6) *NI*(*x*, *y*) denotes the noisy image, and *GWN*(*x*, *y*) stands for the Gaussian white noise of a cell. In this expression “Gaussian” refers to the distribution of the added random values, being a normal distribution with 0 mean and *σ*^2^ variance, while “white” indicates that the stochastic activity of cones is independent of each other. The probability density *p*, of *GWN* can be formulated as:

7 $$p(GWN)=\frac{1}{\sqrt{2\pi {\sigma }^{2}}}\cdot \exp \,(\,-\,\frac{GW{N}^{2}}{2{\sigma }^{2}}).$$

### New neuro-physiological vision model

Our new visual acuity model basically follows the steps described above, but also contains some substantial improvements. Instead of applying elementary monochromatic calculations described by (2), we characterize the complete, polychromatic optical transfer using a physiological eye model. In this way, both ray-tracing and scalar diffraction analysis are taken into account to accurately model the imaging system. Additionally, the schematic eye enables us to make modifications to its structure and analyze the resulting effects. This personalization can be realized in two ways. First, we can make a structurally correct model of a given person’s eye based on biometric measurements (using ultrasonometry, corneal topography etc.), by which it would be possible to design certain visual optical devices (such as IOLs) directly for improved visual acuity. Second, using a structurally average eye model we can customize it by appropriately inserting a given person’s wavefront map *OPD*(*X*, *Y*), measured at a given wavelength and let the model deal with polychromatic aberrations and diffraction. This approach makes it possible to develop e.g. new diagnostic methods by which visual acuity could be objectively determined from a quick wavefront measurement. In this paper we pursue this second way, as it better serves our aims with respect to model calibration (see the “Results” section).

#### Personalized physiological eye model

Our physiologically accurate average eye model is implemented in Zemax OpticStudio^39^. Its structure is kept simple, since individual wavefront aberration is to be introduced to the model directly from measurements. From the many existing schematic eyes, our choice fell on the well-known “historical” Gullstrand Exact (No. I) model^40,41^, which involves only spherical surfaces and simplifies the gradient-index crystalline lens to a central nucleus (core) of high refractive index, surrounded by a cortex of lower refractive index. Since longitudinal chromatic aberration plays important role in human vision^11,21,22^ and the Gullstrand Exact model excludes wavelength dispersion, we replaced its original fixed refractive indices with dispersion formulae based on Atchison and Smith’s measurements^42^. They used the Cauchy formula, but as it was not available in Zemax we fitted their dispersion curves^42^ using the Sellmeier 1 formula, which has an implementation in most optical design software:

8 $${n}^{2}-1=\frac{{K}_{1}{\lambda }^{2}}{{\lambda }^{2}-{L}_{1}}+\frac{{K}_{2}{\lambda }^{2}}{{\lambda }^{2}-{L}_{2}}+\frac{{K}_{3}{\lambda }^{2}}{{\lambda }^{2}-{L}_{3}}.$$

Here *n* denotes the refractive index of the material, *λ* indicates the wavelength in vacuum (given in microns), while *K*_1,2,3_ and *L*_1,2,3_ are coefficients describing a specific material. Their fitted values are summarized in Table 1 for all media of the schematic eye. The result of the fit follows the profile given by Atchison and Smith^42^ with high accuracy: the standard deviation of the refractive indices determined by the Cauchy and the Sellmeier 1 formula being smaller than 0.0017 for all media of the eye model. Polychromatic illumination is weighted as a function of wavelength according to the photopic sensitivity curve of the human eye^3^. In total we apply 24 discrete wavelengths equally distributed across the visible spectral range.

**Table 1:** The numerical values of the *K*_1,2,3_ and *L*_1,2,3_ coefficients used in the Sellmeier 1 formula to describe the wavelength dispersion of the different media of the eye model.

Material	*K*_1_ [−]	*K*_2_ [−]	*K*_3_ [−]	*L*_1_ [*μm*^2^]	*L*_2_ [*μm*^2^]	*L*_3_ [*μm*^2^]
Cornea	8.68·10^−1^	4.60·10^−4^	3.93·10^2^	9.35·10^−3^	1.41·10^−1^	4.41·10^4^
Aqueous	7.61·10^−1^	4.18·10^−4^	5.18·10^4^	1.01·10^−2^	1.42·10^−1^	5.73·10^6^
Lens nucleus	9.42·10^−1^	1.29·10^−3^	4.71·10^4^	1.12·10^−2^	1.32·10^−1^	6.19·10^6^
Lens cortex	1.26·10^−3^	8.87·10^−1^	3.63	1.31·10^−1^	1.15·10^−2^	4.88·10^2^
Vitreous	7.61·10^−1^	2.73·10^−4^	4.30·10^4^	1.01·10^−2^	1.43·10^−1^	5.61·10^6^

**Figure 2:**
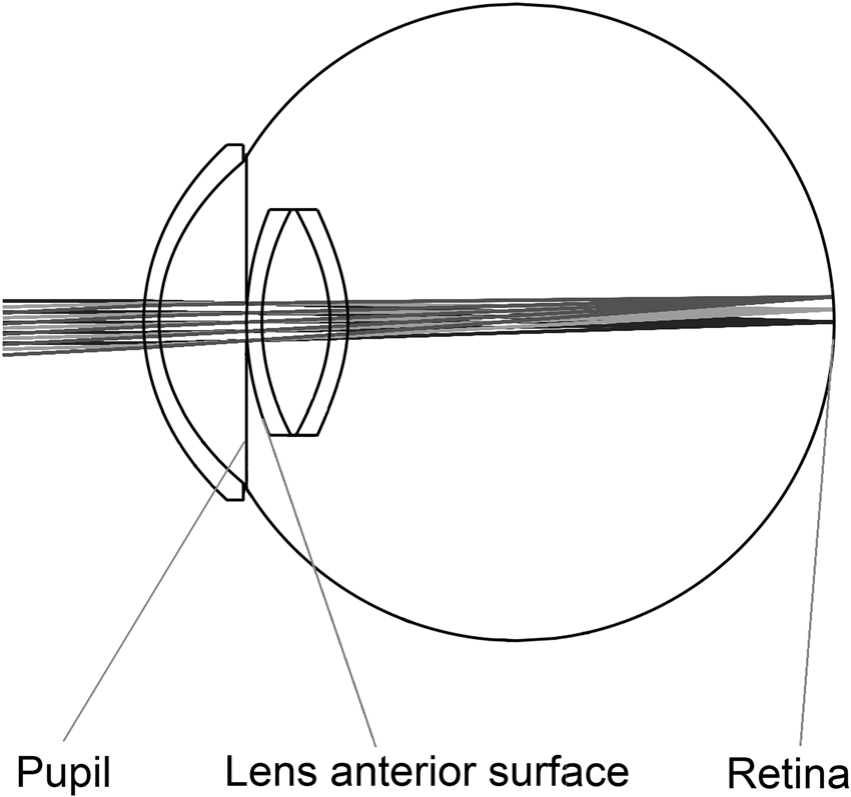
Schematic view of the physiological eye model.

The Styles-Crawford effect (i.e. the directional sensitivity of cones) is taken into account using a Gaussian apodization in the amplitude transmission factor *T*(*X*, *Y*), of the pupil function, see (2). Its single parameter, the 1/*e* radius equals 2.9 mm^40^. The schematic view of our Zemax model is shown in Fig. 2, its mechanical dimensions are presented by Gobbi^40^.

In order to personalize the model, we use the wavefront aberration of a given subject measured by a medical Shack-Hartmann sensor. The outputs of the aberrometer are the coefficients of the Zernike polynomials fitted to the measured wavefront of the subject^11,13–15^. We use a tenth-order expansion, so that *OPD* is given by a set of 64 Zernike coefficients (the zero-order piston, the first-order horizontal and vertical tilts, and also the ${Z}_{2}^{0}$ second-order defocus terms are ignored to exclude instrumental myopia^15^). After a necessary rearrangement (due to the different ordering of Noll’s sequential indices used in Zemax^39^ and OSA/ANSI standard indices used by wavefront sensors^15^), the coefficients can be directly imported to Zemax to characterize the target wavefront. To this end, the first surface of the lens (see Fig. 2) has been modified to be a so-called Zernike-surface, having variable coefficients.

As the first step of personalization, we adjust the entrance pupil diameter *d*, to the value measured by the Shack-Hartmann sensor. Then, we optimize the Zernike coefficients of the lens anterior surface, so that the monochromatic *OPD* equals the measured wavefront shape at the reference wavelength of the Shack-Hartmann sensor, i.e. *λ*_0_ = 555 nm. As a consequence, the model produces exact results at the reference wavelength. Thanks to the close-anatomical structure of the Gullstrand Exact model and the applied Atchison-Smith dispersion formulae, this model allows us to take the effects of chromatic aberrations into account.

After this process of personalization, we change the entrance pupil diameter to the specific value at which the visual acuity of the subject is to be determined. Pupil diameter is a very important parameter, thus we developed a custom arrangement for its real-time measurement during visual acuity tests^43^, see subsection “Experimental setup used for calibration”. For subjects having emmetropic or hypermetropic eyes we optimize the axial retina position for infinite object distance in order to ensure a sharp image. The optimization error function includes the diffraction Modulation Transfer Function, *MTF*, at two distinct spatial frequencies: 14.85 and 22.28 cycles/degree (50 and 75 cycles/mm at the retina, respectively). For subjects with negative refractive error the retina position optimization is performed using an extra paraxial lens (i.e. an ideal model of eyeglasses prescribed for best correction). In order to simulate their uncorrected visual acuity, which is our intention, the paraxial lens has to be removed afterwards. In terms of Zernike polynomials, their ${Z}_{2}^{0}$ coefficient has a negative value.

**Figure 3:**
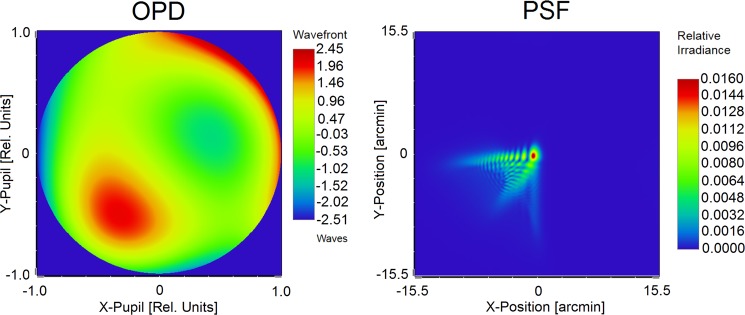
The measured wavefront aberration *OPD*, of subject S. T.’s OD eye and the corresponding polychromatic *PSF* at *d* = 5.0 mm entrance pupil diameter derived from his personalized physiological eye model.

Finally, the polychromatic diffraction *PSF* of the eye is determined and normalized so that its surface integral equals unity. This is an important pre-processing procedure for character recognition^29,30^, that allows for the comparison of results from subjects having different optical aberrations. A representative *OPD* map and the corresponding high-accuracy *PSF*— determined by the personalized model of the subject’s eye at a typical entrance pupil diameter of 5.0 mm— is depicted in Fig. 3.

It can be argued that the effective focal length of our eye model equals the well-known average value of 17.1 mm^22,40,41^, which does not necessarily correspond to that measured on a given person. Having said that the examined letter size, the pupil diameter, and the wavefront aberration are all known in the object space, the focal length of the actual eye cannot affect the image quality in any respect.

The output of the eye model is the numerical matrix representation of the polychromatic diffraction *PSF*. In order to provide accurate angular sampling even for diffraction-limited *PSF*s, we defined the matrices of all filters and images so that the visual angle of one pixel corresponds to Δ*x* = Δ*y* = 5.724 arcsec. Since we implemented Fourier transform using the fast Fourier transform method^44^, FFT, the number of grid points *N*, must be a power of two. In order to ensure that the *PSF* of strongly aberrated eyes can also be evaluated, we set *N* = 1024. From this, the frequency-domain resolution can be calculated as:

9 $$\Delta f=\frac{1}{N\cdot \Delta x}=0.6142\,{\rm{c}}{\rm{y}}{\rm{c}}{\rm{l}}{\rm{e}}{\rm{s}}/{\rm{d}}{\rm{e}}{\rm{g}}{\rm{r}}{\rm{e}}{\rm{e}}.$$

#### Numeric model of neural image processing

The neural part of the model, which imitates the subsequent steps of image processing and character recognition, is implemented in MatLab^45^ to take advantage of its efficient numerical matrix calculations. All operations are accomplished in the frequency domain and the only piece of input data is the polychromatic diffraction *PSF* calculated using Zemax. From this the *OTF* is determined by inverse fast Fourier transform, IFFT, and is applied to the ideal image of the visual target object according to (3). Our target objects are single letters of different sizes, represented by 1024 × 1024 binary matrices. Black pixels of the letter strokes are represented by 0 and white background pixels are indicated by 1.

We use the average *NTF* described by (4) to obtain the post-retinal neural image. In our model, similarly to the *PSF*, the *NTF* is normalized so that the spatial domain surface integral of its Fourier transform equals unity, i.e. *NTF*(*f* = 0) = 1. Due to this normalization, the background value of the filtered images always remains unity. At this point, there is a numerical issue that needs to be mentioned. When a Fourier transform and its inverse are performed one after the other, both calculations have to be accomplished using the same algorithm in order to obtain the correct results. In the present case, we use IFFT for *OTF* calculation, but the *NTF* has not been determined this way. For this reason, we first apply a discrete Fourier transform, DFT, to the *NTF*, then we transform it back by IFFT to obtain the *NTF'*, which we use in our algorithm:

10 $$NT{F}^{{\rm{^{\prime} }}}={\rm{I}}{\rm{F}}{\rm{F}}{\rm{T}}\,[{\rm{D}}{\rm{F}}{\rm{T}}\,[NTF]].$$

**Figure 4:**
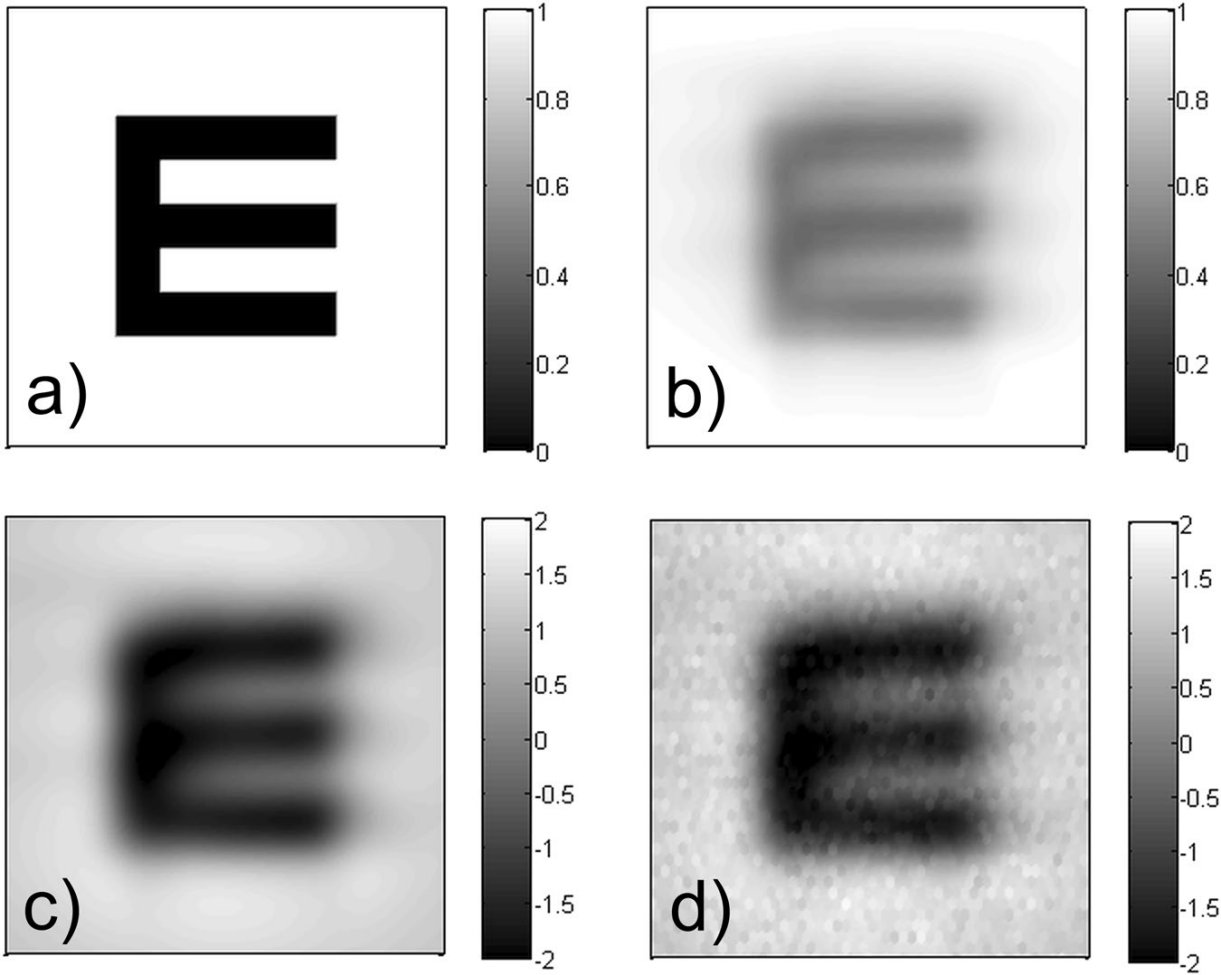
The results of the successive image processing steps of our vision model: (**a**) ideal image of the examined letter, (**b**) retinal image, (**c**) post-retinal neural representation, (**d**) noisy image.

The last step of neural image processing is the calculation of a noisy image, according to (6). In case of high-acuity human vision, one of the most important noise sources is the limited channel capacity of the retinal ganglion cells^17,46^. Thus, we decided to represent neural noise so that it corresponds to the individual cells of the retinal cone lattice, i.e. a hexagonal structure with 120 cycles/degree spatial frequency^10,17,36^. We apply additive Gaussian white noise according to (6) and (7). In order to differentiate between the subjects’ neural sensitivity, and to model temporal changes in their concentration ability, the standard deviation *σ*, of the applied normal distribution is a free parameter of the model that has to be optimized. Since the background of the images is unity, the numerical value of *σ* represents a signal-to-noise ratio relative to the noiseless image. The results of the successive steps of opto-neural image processing are illustrated in Fig. 4. The last sub-image (d) is used as input for the character recognition algorithm.

#### Template-matching model of cortical character recognition

The final phase of our vision model is character recognition. In order to provide a simple algorithm with great descriptive power, like many other authors^18,19,35^, we use template-matching. It quantifies similarity between the examined object and several templates by normalized cross-correlation^18,20,47^. In our model, subjects are considered as ideal observers limited by optical and neural filtering, as well as neural noise, which determines the noisy image as described above. However, the construction of template images requires special consideration.

Despite all distortions being caused by the complex multi-step visual process, people see objects equally sharp in a wide range of environmental conditions. This can be explained by the fact that the brain is able to store and retrieve different filtered images from prior experiences. These images are used as templates, and are compared to the actual noisy image during recognition. According to the literature^19,24,48^, human subjects (and even animals) can learn and memorize the permanent properties of multiple filtered images of an object at different scales and different positions, but cannot learn their variations. Consequently, subjects can form average templates for target objects that had passed through the steps of cortical image processing^9^. Therefore, in our model the template set contains optically and neurally filtered, but *noiseless* images of the same size as the tested image.

We determine the normalized cross-correlation of two matrices using Pearson’s correlation formula^49,50^. Its result is not a single scalar number, but a matrix with a linear size that equals the sum of the original linear sizes less one. Since correlation calculations are highly time-consuming and are called multiple times during a visual acuity simulation, they significantly affect the running time of the algorithm. Therefore, we estimate the lateral extension of the template images by taking two times their second momentum (root-mean-square value), and perform cross-correlation only on matrices covering this area. In this way, the correlation matrix size depends on the actual image size, but is strictly smaller than 200 × 200 (cf. the original 1024 × 1024), which boosts the calculation significantly. Furthermore, by cutting off the constant background borders, we eliminate the possibility of fake high correlation peaks corresponding to shifted positions where only the edges of the two matrices overlap. Finally, the correlation value *ρ*, is determined from the maximum element of the calculated cross-correlation matrix, which ensures translation invariance for recognition^23,25,48^.

In the case of a tested letter, the template-matching algorithm determines the above-described correlation value for all possible templates and sorts them in descending order to find the maximum: *ρ*_*max*_. Though simple recognition models define the response directly by *ρ*_*max*_^7,9^, we noticed that during visual acuity tests the subjects often become hesitant near the recognition threshold and start guessing from multiple choices. This suggests that differences below a certain threshold cannot be distinguished^5,51^. In order to reconstruct the decision-making method accordingly, we defined a discrimination range *δρ*, in which the identification of any differences is not possible. We consider indistinguishability bound to an absolute limit, thus we have determined *δρ* as a constant value, which is another free parameter of our visual acuity model. Accordingly, the output of our recognition process is a set of potential guesses corresponding to the correlation values within the [*ρ*_*max*_ − *δρ*; *ρ*_*max*_] discrimination range. In this way, the vision model has only two independent parameters: *σ* and *δρ*, which we optimized based on our measurements, see “Experimental setup used for calibration” and “Results”.

#### Determination of the visual acuity value using correlation-based scoring

We have formerly introduced a correlation-based scoring scheme to reduce the statistical error of visual acuity tests^52,53^. As we make extensive use of it in the vision model, a brief summary is given below and some differences from the standard probability-based scoring are expressed. Further details and deeper explanations are presented in our former papers^52,53^.

During conventional visual acuity measurements, the examiner only registers whether the optotypes are identified correctly or not, i.e. the mere fact of recognition is tested. Thus, the responses are represented as binary scores, where 1 represents a correct recognition and 0 indicates a mistake. However, in some cases the examiner omits minor errors (e.g. misidentifying C as O, or R as P)^5^, which suggests that human visual perception is more complex than a simple binary scheme. In other words, whenever the subject makes a mistake, they might see some features of a specific letter, so it is worth characterizing how bad or good the guess is. Consequently, we have previously introduced a new quantity called Optotype Correlation, *OC*, as a measure of character similarity^52,53^. The quantification is based on Pearson’s normalized cross-correlation^50^ calculated on the non-distorted original images of letters. For a given character pair we take the correlation peak, which results in a single real number over the [−1; 1] interval. From this, *OC* is obtained after a linear transformation, so that the expected value of the statistics of *OC* for two *randomly selected* letters equals 1/26 (because we use all 26 letters of the English alphabet in our examinations), and *correct recognitions* are always represented by 1. In this way, *OC* is directly comparable to the conventional binary scheme of true/false identifications, but provides more information about the specific response. The *OC* values for all potential combinations of displayed-guessed characters can be tabulated as a two-dimensional array, the so-called *OC* matrix. This is similar to confusion matrices (used both in ophthalmology^51^ and machine learning^26^ to quantify the probability of misidentifications), since letter pairs with higher *OC* values are more likely to be confused.

**Figure 5:**
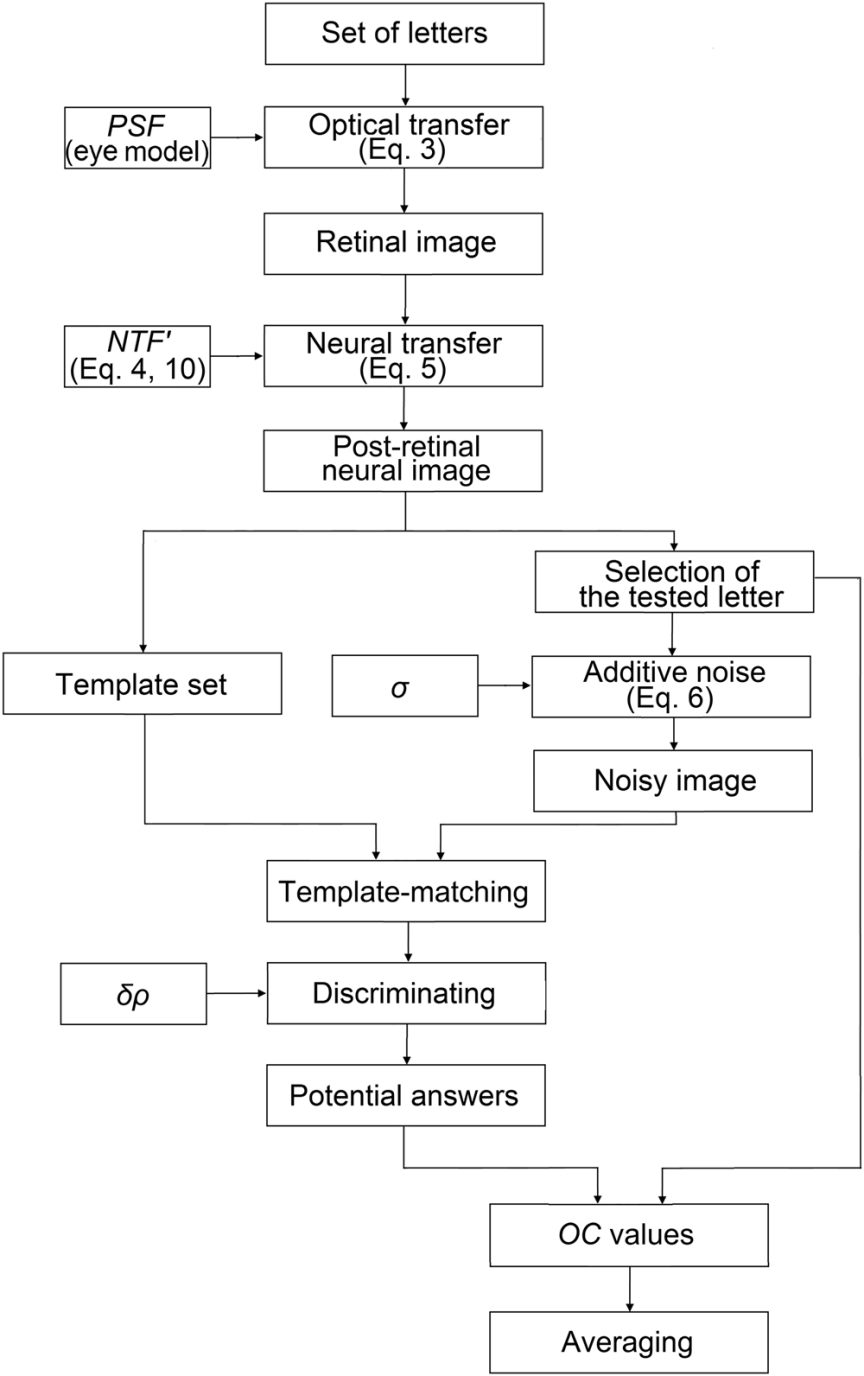
Workflow of our vision model describing the identification of one single letter. Main input parameters are indicated.

The discrimination range may contain one or more potential responses in case of each single tested letter. Accordingly, our recognition model calculates the *OC* value for all these guesses and yields the output for the tested letter as their mean. In such ambiguous cases the averaging step represents the hesitation of the subject by summarizing the possible results of multiple acuity tests performed subsequently, thereby decreasing uncertainty error. The workflow of the entire process for the identification of *one single letter*, together with its main parameters, is depicted in Fig. 5.

When testing *multiple letters*, the standard method characterizes perception quality at a given letter size by the recognition probability *P*. Instead of this, we have proposed a new quantity, the Rate of Recognition, *RR*, that better describes visual quality, but is still directly comparable to recognition probability^52,53^. For a fixed letter size *s* (i.e. the actual line of the ETDRS chart), *RR* is defined as the average *OC* value of the tested-identified character pairs:

11 $$RR\equiv \overline{OC}{|}_{s=const.}.$$

After scoring the individual identifications and evaluating their distribution, the last step of our vision model is to determine the visual acuity value of the examined eye. In our former paper^52^ we concluded that thresholding the visual psychometric function of a given subject provides the most precise acuity result without any bias^52,54^. If we express the letter size *s*, in logMAR units:

12 $$s\equiv {\log }_{10}(\alpha ),$$

where *α* denotes the visual angle of the stroke width of letters, then the $s\mapsto RR(s)$ relation represents the psychometric function of vision^55–57^. We describe its profile by the sigmoid-shape logistic function *L*(*s*), which is the most common two-parameter curve used to approximate any psychometric function^55,56,58^. We linearly transform the logistic function to ensure its limits correspond to the theoretically expected *RR* values, according to the total number of possible responses^57^ (i.e. all 26 letters of the English alphabet). Its mathematical formula is described by the following equation:

13 $$L(s)=\frac{25}{26}\cdot \frac{1}{1+\exp \,(4k(s-{s}_{mp}))}+\frac{1}{26},$$

where *s*_*mp*_ sets the midpoint position of the sigmoid and *k* determines the steepness of the curve at this point.

Based on the calculated points *RR*(*s*), of the given subject, we determine the two parameters of the psychometric curve *L*(*s*), by logistic regression. Then, the visual acuity value *V*, is given by that letter size *s*_0_, at which the *RR* value equals the correlation threshold, *RR*_0_:

14 $$L({s}_{0})={RR}_{0}{|}_{k,{s}_{mp}}\Rightarrow V\equiv {s}_{0}.$$

**Figure 6:**
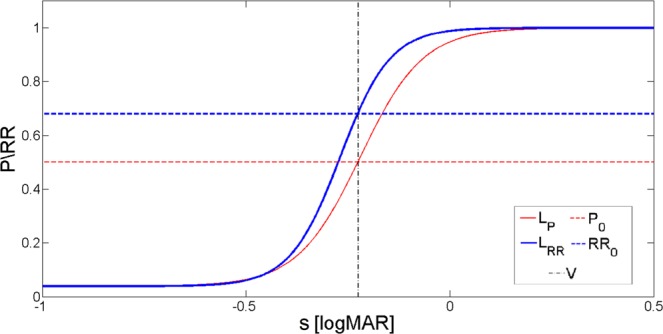
Average psychometric functions based on our high-precision measurements both in cases of probability and correlation-based scorings, together with the corresponding thresholds: *P*_0_ = 0.5 and *RR*_0_ = 0.68^52^.

This method follows the idea of conventional visual acuity measurements evaluated using curve-fitting in terms of recognition probability, where only the probability threshold *P*_0_ = 0.5, of the ICO standard^1,2^ has to be replaced with an adequate value of *RR*_0_. We have formerly calibrated the threshold level by our high-precision measurements taken under special laboratory conditions: *RR*_0_ = 0.68 (for details see^52^). Figure 6 depicts the concept of curve-fitting-based evaluation using the average psychometric curves determined in our previous paper^52^ (*L*_*P*_: *s*_*mp*_ = −0.214 logMAR, *k* = 3.306 logMAR^−1^, and *L*_*RR*_: *s*_*mp*_ = −0.269 logMAR, *k* = 4.278 logMAR^−1^). Differences between the probability and correlation-based scoring methods are clearly visible.

### Experimental setup used for calibration

So that we could provide accurate data for the calibration of the two free neural parameters of our vision model and demonstrate its operation, we performed visual acuity tests on real subjects. During these trials we used a special computer-controlled measuring system^52^ that is much more precise than standard visual acuity tests, thanks to the applied correlation-based scoring, increased number of tested characters, and denser letter-size sampling. Below a brief description of our measurement is given and some differences compared to conventional acuity tests are expressed. Further details and explanations are presented in our previous papers^52,53^.

In order to reduce statistical error, we examined not only five-letter lines printed on a standard ETDRS chart, but the complete extended Sloan font type^59^ comprising of all 26 letters of the English alphabet at each size. We used a Dell U2312HMT in-plane-switching LCD monitor for the visualization of optotypes. As there was not enough space to display all 26 letters on a single line, the black characters were presented individually, one after another in random order, in the middle of a solid white background. This is exactly the same way as our model simulates it. The subjects were told beforehand that they would be presented only with letters. Accordingly, in our model the template set comprised of the 26 letters of the English alphabet, each being a possible response. We examined 14 letter sizes ranging from 0.3 to −0.35 logMAR with steps of 0.05 logMAR. During the trial the subject verbally identified each displayed optotype and the examiner promptly typed the response on a keyboard. Right after the measurement the controller software automatically scored the test using *OC*, evaluated the collected data (11), and performed logistic regression (13) to determine the visual acuity value (14). A measurement trial lasted 30…40 minutes due to the large number of tested letters and dense size sampling.

It has long been known that pupil size significantly influences visual acuity^1,3,38,60^. Based on our simulations, 1.1 mm alteration in the pupil diameter could cause 0.05 logMAR change in the acuity value, which confirmed this observation. Accordingly, we paid special attention to control the surrounding illumination and to measure pupil size. As the human pupil diameter is larger under mesopic than photopic conditions, which emphasizes the imaging errors of the eye^1,3,11,60^, we implemented our measurement in a darkened room with an illuminance level of around 5…10 lux. Furthermore, during the trial the subject watched the monitor with one eye, while the other was covered with a transparent but opaque shield (i.e. a diffuser) to keep the pupil size at the specific value it was adapted to with both eyes open. In addition, we continuously measured the pupil diameter using our custom-made far-field machine vision system^43^. We applied infrared illumination to provide appropriate lighting conditions for high-resolution images and so as not to affect the dark-adapted pupil size. We monitored (but not controlled) the pupil using a digital camera and determined the accurate pupil diameter by our adaptive circular-Hough-transform-based algorithm corrected for magnification error^43^. The pupil size measurement and the acuity test were synchronized, hence we knew the exact pupil diameter *d*_*m*_, corresponding to each recorded response.

In order to simulate exactly the above-described acuity tests with our vision model, the wavefront aberration of the eyes of each individual subject was measured using a clinical Shack-Hartmann sensor (WASCA Asclepion Zeiss Wavefront Analyzer, SW 1.41.6; Carl Zeiss Meditec AG, Germany). The outputs of the wavefront sensor were the pupil diameter *d*_*w*_, recorded at the wavefront measurement, and the coefficients of the Zernike polynomials, which were needed to personalize the Zemax eye model. For the calculation of the polychromatic diffraction *PSF* we applied the pupil diameter *d*_*m*_, measured for each tested letter separately during the acuity trials. In our simulation we considered exactly the same letter sizes and character set as in our measurement, so that we could reconstruct the monocular visual acuity value of the examined subjects as precisely as possible.

We carried out our demonstrative trials through the cooperation of eight young subjects (ages of 22…40), none of whom wore prescription eyeglasses. Their visual acuity was between 0…−0.3 logMAR, close to the range of normal vision (−0.1…−0.2 logMAR^3,61^). They were selected to be close-emmetropic so that they could always sharply focus on the eyechart. The results of their wavefront measurement, including the pupil diameter *d*_*w*_, are summarized in Table 2. The refractive error *RE*, and the astigmatism *Cyl*, of the subjects’ eyes were measured using a TopCon autorefractor. Although we gathered all data for each of the subjects’ eyes, we only considered their right eyes (OD) in our analyses, as an individual’s eyes are often strongly correlated^62,63^ and do not, therefore, provide additional information.

**Table 2:** Summary of the wavefront aberration measurements: the refractive error *RE*, the astigmatism *Cyl*, the pupil diameter *d*_*w*_, and the second, third, and fourth-order Zernike coefficients of the applied tenth-order expansion (${Z}_{2}^{0}$ defocus term is considered to be zero, except for subject K. M. for whom ${Z}_{2}^{0}=-2.24\,{\lambda }_{0}$). *λ*_0_ = 555 nm.

Subject	*RE* [D]	*Cyl* [D]	*d*_*w*_ [mm]	${{\bf{Z}}}_{2}^{-2}$ [*λ*_0_]	${{\bf{Z}}}_{2}^{2}$ [*λ*_0_]	${{\bf{Z}}}_{3}^{-3}$ [*λ*_0_]	${{\bf{Z}}}_{3}^{-1}$ [*λ*_0_]	${{\bf{Z}}}_{3}^{1}$ [*λ*_0_]	${{\bf{Z}}}_{3}^{3}$ [*λ*_0_]	${{\bf{Z}}}_{4}^{-4}$ [*λ*_0_]	${{\bf{Z}}}_{4}^{-2}$ [*λ*_0_]	${{\bf{Z}}}_{4}^{0}$ [*λ*_0_]	${{\bf{Z}}}_{4}^{2}$ [*λ*_0_]	${{\bf{Z}}}_{4}^{4}$ [*λ*_0_]
G. A.	0.0	−0.5	4.5	−1.32·10^−1^	−1.59·10^−1^	−4.15·10^−2^	−4.09·10^−2^	−3.32·10^−2^	7.52·10^−2^	4.86·10^−2^	4.02·10^−2^	2.98·10^−2^	−6.97·10^−2^	7.45·10^−2^
M. T.	0.25	0.5	6.2	−3.57·10^−2^	−2.54·10^−1^	−2.68·10^−1^	−3.81·103^−1^	6.28·10^−2^	1.40·10^−1^	−4.13·10^−2^	5.40·10^−3^	9.23·10^−2^	6.07·10^−2^	−7.46·10^−2^
P. B.	0.5	0.5	5.8	4.34·10^−1^	1.91·10^−1^	3.43·10^−1^	−2.09·10^−2^	−7.65·10^−2^	−4.06·10^−2^	9.23·10^−2^	−3.86·10^−2^	2.27·10^−1^	1.36·10^−1^	2.90·10^−2^
S. T.	0.25	0.0	5.4	1.42·10^−1^	−1.39·10^−1^	−6.18·10^−2^	2.02·10^−1^	2.93·10^−1^	8.87·10^−2^	−4.35·10^−2^	5.55·10^−3^	−5.71·10^−2^	5.94·10^−2^	−3.04·10^−2^
U. F.	0.25	−0.25	2.9	−4.63·10^−3^	6.81·10^−2^	−1.22·10^−2^	1.70·10^−2^	1.05·10^−2^	−4.59·10^−3^	1.45·10^−3^	4.80·10^−3^	5.53·10^−3^	3.67·10^−3^	2.19·10^−3^
K. M.	−0.5	−1.25	6.3	1.82·10^−1^	1.15	−1.63·10^−2^	−7.06·10^−2^	−1.06·10^−1^	−1.20·10^−2^	4.61·10^−3^	4.35·10^−2^	9.13·10^−2^	1.20·10^−2^	6.06·10^−2^
S. O.	1.5	0.0	6.2	3.93·10^−1^	−1.93·10^−1^	−3.31·10^−1^	1.45·10^−1^	−6.66·10^−3^	1.42·10^−1^	−3.68·10^−2^	6.14·10^−2^	1.98·10^−1^	−4.30·103^−2^	−1.05·10^−2^
G. T.	0.0	−0.25	6.2	2.11·10^−1^	2.21·10^−2^	−3.29·10^−2^	−2.31·10^−1^	5.65·10^−2^	5.84·10^−2^	−4.91·10^−2^	4.53·10^−2^	1.21·10^−1^	−5.39·10^−2^	−7.90·10^−3^

## Results

We calibrated the free neural parameters of our vision model by comparing the predictions of acuity simulations to the outcomes of the above-described visual acuity measurements. Using extremum detection over an appropriate parameter space we sought for that specific *σ* and *δρ* pair that minimized the root-mean-square difference Δ*RR*, between the simulated *RR*_*s*_(*s*_*i*_), and the measured *RR*_*m*_(*s*_*i*_), rate of recognition values (note: the same letter sizes were tested). For each given subject the fitting error takes the form of the following expression:

15 $$\Delta R{R}^{2}\equiv \mathop{\sum }\limits_{i=1}^{14}\,{(R{R}_{s}({s}_{i})-R{R}_{m}({s}_{i}))}^{2},$$

where *i* indicates the index of the letter sizes. This method provides a more comprehensive error figure than the pure difference in visual acuity, since that is only a single scalar number^58^. We performed the calibration for each person examined individually and also for the entire subject pool using common *σ* and *δρ* parameters.

### Individual calibration of model parameters

**Figure 7:**
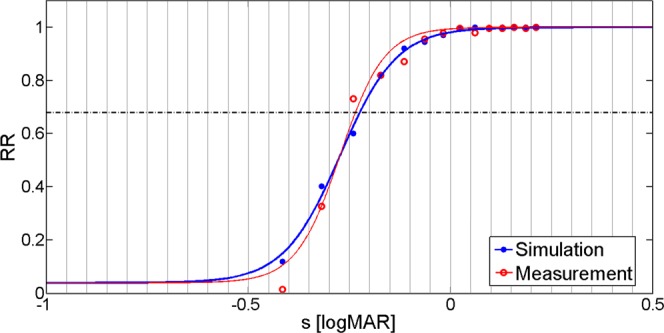
Visual acuity measurement and simulation results for subject S. O.’s OD eye. Filled and hollow circles represent registered values, while continuous curves show the psychometric functions obtained through logistic regression.

The correct operation of our vision model can be best investigated by optimizing *σ* and *δρ* for all tested subjects *independently* of each other. We completed this *individual* calibration by performing simulations in the parameter space of *σ* = 0…0.15; *δρ* = 0…0.005, and compared the predictions of the model to the outcome of the measurement person by person. In order to avoid any bias, we determined both the simulated and the measured visual acuity values according to (14). A representative example for the measured/simulated *RR* values and the fitted psychometric curves is depicted in Fig. 7. The measured and the best-fit visual acuity values, together with the optimized parameters and the corresponding fitting error are presented in Table 3 for each subject.

**Table 3:** Summary of our individual calibration results: measured pupil diameter *d*_*m*_, averaged for a given subject, measured *V*_*m*_, and best-fit simulated *V*_*bf*_, visual acuity values, and fitting error Δ*RR*.

Subject	*d*_*m*_ [mm]	*V*_*m*_ [logMAR]	*V*_*bf*_ [logMAR]	Δ*RR* [−]	*σ* [−]	*δρ* [−]
G. A.	4.6	−0.27	−0.26	0.025	0.075	0.002
M. T.	5.6	−0.22	−0.21	0.060	0.150	0.002
P. B.	4.8	−0.18	−0.16	0.047	0.125	0.003
S. T.	5.6	−0.25	−0.23	0.050	0.100	0.002
U. F.	3.8	−0.31	−0.30	0.033	0.150	0.003
K. M.	6.0	−0.09	−0.07	0.047	0.125	0.002
S. O.	5.0	−0.24	−0.23	0.039	0.150	0.003
G. T.	6.0	−0.25	−0.25	0.034	0.075	0.002

The optimized model parameter values are listed below *σ* and *δρ*.

The average difference between the best-fit simulated *V*_*bf*_, and the measured *V*_*m*_, visual acuity values is 0.010 logMAR, which approximates the systematic error of individual best-fit simulations, while the standard deviation equals 0.0055 logMAR. The sum of these two estimates the best achievable residual of our simulation over the calibration group: 0.016 logMAR. A comparative error analysis will be presented in the “Discussion” section, revealing that this value approximately equals the uncertainty of our high-precision acuity measurements^52^. This implies that the precision of the simulations is limited by the reference measurements, and demonstrates that proper adjustment of the model is feasible with only two free neural parameters.

### Investigation of sensitivity to changes in model parameters

In order to examine the required complexity of our vision model, and to assess the role of optical effects and neural transfer, we subsequently performed additional simulations by omitting the *PSF* and the *NTF*. Based on the results we conclude that the free neural parameters cannot be calibrated if any part of the model is discarded. This confirms that both optical filtering and neural transfer are key elements of the simulation to accurately reconstruct visual acuity. Similarly, we performed the individual calibration by neglecting either *σ* or *δρ*, but in all cases the fitting error Δ*RR*, and the average difference between simulated and measured acuity values were larger than with both parameters optimized simultaneously.

We evaluated the reliability of our model by carrying out an inverse-sensitivity analysis. In this simulation we sought the change of construction parameters necessary to cause Δ*V* = 0.05 logMAR alteration in the visual acuity value. For this purpose, we varied the entrance pupil diameter *d*, and the two neural parameters. We also examined the effect of changes in the wavefront shape by introducing some artificial refractive error *RE*, corresponding to a small amount of defocus. The results of the analysis are presented in Table 4.

**Table 4:** Results of the inverse-sensitivity analysis.

Parameter	Average value ± standard deviation	Change required for Δ*V* = 0.05 logMAR
Pupil diameter (*d*)	5.2 ± 0.77 mm	1.1 mm
Refractive error (*RE*)	0 D*	<0.25 D
Additive Gaussian white noise (*σ*)	0.12 ± 0.03	0.15
Discrimination range (*δρ*)	0.0024 ± 0.0005	0.0012

Averages and standard deviations are given for the calibration group. *It is practically zero, since almost all our subjects could naturally focus on the eyechart.

These results confirm our expectation, that the vision model is highly sensitive to the optical input parameters. This means that their precise knowledge is essential to accurately simulate the acuity value of a specific eye. Moreover, it is observable that the model is considerably less sensitive to the adjustment of the neural parameters, which implies that it may be possible to achieve promising results by using *average* neural features together with *personalized* wavefront/pupil data. In this way, the lengthy individual calibration process might be eliminated. An investigation of a potential average neural model is presented below.

### Optimum neural parameters for the average visual system

In order to determine the parameters of the average neural model, we performed global optimization considering all subjects *simultaneously*. We determined the fitting error Δ*RR*_*ave*_, again using the root-mean-square deviation between the simulated and the measured *RR* values, but this time we calculated it for *all* examined eyes and analyzed their *average* over the subjects. We found the optimum parameters of the average neural model to be *σ*_*ave*_ = 0.10 and *δρ*_*ave*_ = 0.0025. These are close to the mean of the individual best-fit parameters listed in Table 3, however provide more precise estimates. The predictions of the average model, together with the outcomes of the measurement and the resulting fitting errors are presented in Table 5.

**Table 5:** Comparison of the average neural model and the measurement: measured *V*_*m*_, and simulated *V*_*ave*_, visual acuity values (*σ*  = 0.10 and *δρ* = 0.0025).

Subject	*V*_*m*_ [logMAR]	*V*_*ave*_ [logMAR]	Δ*RR*_*ave*_ [−]
G. A.	−0.27	−0.22	0.065
M. T.	−0.22	−0.21	0.067
P. B.	−0.18	−0.19	0.064
S. T.	−0.25	−0.21	0.070
U. F.	−0.31	−0.33	0.049
K. M.	−0.09	−0.05	0.069
S. O.	−0.24	−0.26	0.070
G. T.	−0.25	−0.21	0.069

Δ*RR*_*ave*_ indicates the root-mean-square difference between simulated and measured *RR* values.

The average difference between the *V*_*ave*_ simulated and *V*_*m*_ measured visual acuity values is 0.013 logMAR (systematic error), while the standard deviation of the differences equals 0.032 logMAR (uncertainty). The sum of these two estimates the residual of our simulation over the calibration group: 0.045 logMAR, which is certainly larger than the residual of the best-fit model, as expected. Based on our detailed comparative error analysis to be presented in the next section, we conclude that the residual of our simulations using the average neural model over the calibration group is smaller than the error of conventional five-letter line-assignment-based visual acuity tests^3,54,64^.

Due to the small number of participants involved, we can only present the behavior of our model over the calibration group. Further experiments performed on an extended subject pool, including wider age bracket and participants with certain vision loss, are required to conclude general statements about its eventual accuracy.

## Discussion

The accuracy of visual acuity simulations is affected by both systematic and random errors. The source of random error is twofold. First, it comes from neglecting the fine individual characteristics of cortical character recognition, such as learning capabilities, effects of fatigue, mood etc., that may even change over time. Second, it occurs during the simulation, since the additional Gaussian white noise implemented in the model represents a statistical quantity. In order to determine its effect on repeatability, we performed five separate simulations with the same parameter settings and determined the standard deviation of the resulting visual acuity values, in the same way as ophthalmic scientists check test-retest-variability^54,64^. Based on the results, this constituent of random error proved to be smaller than 0.0045 logMAR. This value is very close to the random error of our individual simulation results, providing a reasonable explanation for that and is negligible for all practical purposes.

Behind systematic errors there are those simplifications that we applied when constructing the model. In order to verify the relevance of our assumptions, and to assess their effects on accuracy, we compared our model to a scheme presented in the literature. Since Watson and Ahumada’s model^7^ is the closest to our work, we chose it as a basis for our comparison. From their set of remarkably complex and comprehensive models, we selected that version which agrees with ours in every aspects— except for the optical model of the eye and the decision-making step of the recognition process. To implement their approach, we started from our own vision model (*M*0) and then simply eliminated the differences one after the other. First, we changed the discrimination range to *δρ* = 0, in which case the recognized template is determined simply by the highest cross-correlation value *ρ*_*max*_ (*M*1). Then, we discarded the effects of chromatic aberration by calculating optical transfer from the monochromatic *PSF* (*M*2), and finally we applied a fix pupil diameter *d* = 6 mm, across the entire model (*M*3). This last version corresponds exactly to the model described by Watson and Ahumada^7^. In order to achieve the best comparison of methods, we determined the optimum neural parameters for each subject independently. For the sake of consistency with the other parts of this paper, visual acuity values were evaluated using our correlation-based scoring scheme^52,53^ and logistic regression for all *M*0…*M*3 models. The statistics of neural parameters and fitting errors is presented in Table 6.

**Table 6:** Comparison of simulations performed using modified vision models (for details see the text).

Method	*V* [logMAR]	Δ*RR* [−]	*σ* [−]	*δρ* [−]
*M*0	−0.21 ± 0.07	0.042 ± 0.011	0.12 ± 0.03	0.0024 ± 0.0005
*M*1	−0.23 ± 0.08	0.049 ± 0.016	0.24 ± 0.06	0
*M*2	−0.23 ± 0.07	0.052 ± 0.019	0.31 ± 0.12	0
*M*3	−0.21 ± 0.07	0.079 ± 0.067	0.34 ± 0.10	0

*M*0 refers to our solution, and *M*3 reconstructs the scheme presented by Watson and Ahumada^7^. All indicated values are averages ± standard deviations calculated over the calibration group.

Based on the fitting error Δ*RR*, we conclude that the subsequent *M*0 → *M*1 → *M*2 → *M*3 modifications of the model steadily deteriorate the goodness of fit to the reference measurements. The Δ*RR* value of *M*3 (which reconstructs the model presented by Watson and Ahumada^7^) is twice as large as that of *M*0, justifying our decision to include chromatic aberrations, discrimination range, and precise pupil diameter in the model. Additionally, the average neural noise parameter *σ*, increases with the modifications, which indicates that additive noise gradually takes over the role of other factors that affect image quality, as they are eliminated step by step from the system. The noise of 0.34 in the case of *M*3 is surprisingly close to the value presented by Watson and Ahumada^7^. However, according to the literature^46,65^, *σ* is somewhere between 0.03 and 0.08, to which our value in *M*0 is much closer.

The fitting error is perfectly suitable for the theoretical comparison of different models. However, from the point-of-view of applicability, our simulation has to be compared to other similar methods and real acuity measurements via the Δ*V* value, i.e. the sum of systematic and random errors. Correspondingly, the average residual of our calibration group, together with the accuracy of the Watson-Ahumada model^7^, our high-precision measurements^52^, and conventional visual acuity tests^51,54,64^ are summarized in Table 7. For the sake of completeness, we also share the duration of the analyses. Simulations were performed on a virtual PC with Intel Xeon E5 4620 processor (4 cores, 8 threads, 2.2 GHz) and DDR-3 RAM (1333 MHz) of a Dell PowerEdge R820 four-socket server.

**Table 7:** Accuracy (residual) and duration of different methods used to determine visual acuity.

	Method	Δ*V* [logMAR]	*t* [min]
Simulation	Individual best-fit neural parameters	0.016	≈200*
	Average neural parameters	0.045	15…20
	Watson and Ahumada model^7^	0.056…0.070	*n*/*a*
Measurement	Correlation-based scoring, 26 letters/size^52^	0.017	30…40
	Correlation-based scoring, 5 letters/size^52^	0.053	2…3
	Conventional line-assignment-based ETDRS test^51,54,64^	0.06…0.15	1…2

*Optimization time included.

Based on the results, the personalized best-fit simulation provides approximately the same residual over the calibration group as our high-precision measurement using all 26 letters at each letter size and correlation-based scoring. However, personal optimization of the simulation parameters requires a substantial amount of computation time. The residual of simulations using the average neural model is comparable to the accuracy of our improved-precision measurements taken with five Sloan characters per line and correlation-based scoring^52^. This residual is still much less than the error of standard clinical line-assignment-based ETDRS trials^51,54,64^. Its 15…20 minutes duration may be too lengthy for certain applications, thus it needs to be reduced through further software/hardware optimization.

After a subsequent calibration to be conducted on a broader population sample— including older subjects as well as those with certain loss of vision— our long-term goal is to further refine the model such that it can be used to make efficient program extensions for standard optical design software. In this way, the design of visual optical devices (such as IOLs) directly for improved visual acuity would become possible. For example, by applying a more sophisticated eye scheme (i.e. one including topographic corneal data and biometric ocular dimensions) our simulations could estimate the post-operative visual acuity of patients destined for cataract surgery. Since our method apparently works fine with ocular wavefront-aberration measurements, it might also be translated to clinical practice. With its help new objective tests could be developed to determine the visual acuity in cases where traditional means are not feasible (e.g. testing illiterate adults or preschool children, etc.).

## Data Availability

The datasets generated and analyzed in the study are available from the corresponding author on reasonable request.
