# Author Correction: Simulation of visual acuity by personalizable neuro-physiological model of the human eye

**DOI:** 10.1038/s41598-019-54236-5

**Published:** 2019-11-22

**Authors:** Csilla Fülep, Illés Kovács, Kinga Kránitz, Gábor Erdei

**Affiliations:** 1 Department of Atomic Physics, Budapest University of Technology and Economics, H-1111 Budapest, Hungary; 2 Department of Ophthalmology, Semmelweis University, H-1085 Budapest, Hungary; 3 Department of Ophthalmology, Weill Cornell Medical College, New York City, NY USA

Correction to: *Scientific Reports* 10.1038/s41598-019-44160-z, published online 24 May 2019

The original version of this Article omitted an affiliation for Illés Kovács. The correct affiliations for Illés Kovács are listed below:

Department of Ophthalmology, Semmelweis University, H-1085, Budapest, Hungary

Department of Ophthalmology, Weill Cornell Medical College, New York City, NY, USA

This has now been corrected in the HTML and PDF versions of this Article.

